# Effects of egg-adaptation on receptor-binding and antigenic properties of recent influenza A (H3N2) vaccine viruses

**DOI:** 10.1099/jgv.0.000457

**Published:** 2016-06-15

**Authors:** Lauren Parker, Stephen A. Wharton, Stephen R. Martin, Karen Cross, Yipu Lin, Yan Liu, Ten Feizi, Rodney S. Daniels, John W. McCauley

**Affiliations:** ^1^​ The Francis Crick Institute, Mill Hill Laboratory, The Ridgeway, London, NW7 1AA, UK; ^2^​ Formerly Divisions of Virology, MRC National Institute for Medical Research, The Ridgeway, Mill Hill, London, NW7 1AA, UK; ^3^​ Physical Biochemistry, MRC National Institute for Medical Research, The Ridgeway, Mill Hill, London, NW7 1AA, UK; ^4^​ Department of Medicine, Glycosciences Laboratory, Imperial College London, Du Cane Road, London, W12 0NN, UK

## Abstract

Influenza A virus (subtype H3N2) causes seasonal human influenza and is included as a component of influenza vaccines. The majority of vaccine viruses are isolated and propagated in eggs, which commonly results in amino acid substitutions in the haemagglutinin (HA) glycoprotein. These substitutions can affect virus receptor-binding and alter virus antigenicity, thereby, obfuscating the choice of egg-propagated viruses for development into candidate vaccine viruses. To evaluate the effects of egg-adaptive substitutions seen in H3N2 vaccine viruses on sialic acid receptor-binding, we carried out quantitative measurement of virus receptor-binding using surface biolayer interferometry with haemagglutination inhibition (HI) assays to correlate changes in receptor avidity with antigenic properties. Included in these studies was a panel of H3N2 viruses generated by reverse genetics containing substitutions seen in recent egg-propagated vaccine viruses and corresponding cell culture-propagated wild-type viruses. These assays provide a quantitative approach to investigating the importance of individual amino acid substitutions in influenza receptor-binding. Results show that viruses with egg-adaptive HA substitutions R156Q, S219Y, and I226N, have increased binding avidity to α2,3-linked receptor-analogues and decreased binding avidity to α2,6-linked receptor-analogues. No measurable binding was detected for the viruses with amino acid substitution combination 156Q+219Y and receptor-binding increased in viruses where egg-adaptation mutations were introduced into cell culture-propagated virus. Substitutions at positions 156 and 190 appeared to be primarily responsible for low reactivity in HI assays with post-infection ferret antisera raised against 2012–2013 season H3N2 viruses. Egg-adaptive substitutions at position 186 caused substantial differences in binding avidity with an insignificant effect on antigenicity.

## Introduction

Influenza A virus (IAV), subtype H3N2, causes seasonal human influenza and is included in trivalent and tetravalent vaccines containing H1N1, H3N2 and influenza B virus components. Influenza viruses undergo antigenic drift by mutation of the haemagglutinin (HA) gene, encoding the major protein target for immune responses, to evade pre-existing immunity. Accumulation of these mutations can result in the emergence of antigenically-distinct groups if certain amino acid substitutions are introduced in the HA glycoprotein. To ensure vaccines are as effective as possible, global surveillance and monitoring of circulating wild-type viruses has been carried out since 1948 under the auspices of WHO to monitor influenza virus evolution, and vaccine composition is reviewed by WHO twice annually.

Most influenza viruses used for vaccines have been propagated in hens’ eggs. However, cultivation of clinical samples containing human influenza viruses in eggs can select for virus variants (Robertson* et al.*, 1987; Robertson* et al.*, 1985; Schild* et al.*, 1983; Wang* et al.*, 1989) and these egg-propagated viruses differ from the IAV in clinical specimens to a greater degree than viruses cultivated in tissue culture cells (Katz* et al.*, 1990; Katz & Webster, 1992; Meyer* et al.*, 1993; Rocha* et al.*, 1993; Schild* et al.*, 1983). Cell culture-based influenza vaccines from qualified Vero and MDCK cell lines have been developed as summarised in (Donis* et al.*, 2014; Perdue* et al.*, 2011) and have regulatory approval in the USA and some European countries (Barrett* et al.*, 2013; Doroshenko & Halperin, 2009). However, licensed human seasonal cell-propagated influenza vaccines are derived from an egg-isolated virus. Recent developments show that candidate vaccine viruses can be designed using genetic sequence data and generated rapidly in MDCK cells to produce mimics of circulating viruses (Dormitzer , 2015; Dormitzer* et al.*, 2013). Nevertheless, the vast majority of human influenza vaccines continue to be propagated in eggs to generate high-yield, reassortant, candidate vaccine viruses (CVV).

Virus adaptation to propagation in eggs usually results in the introduction of amino acid (AA) substitutions in the HA glycoprotein. Due to the close proximity of the receptor binding site (RBS) and areas targeted by virus neutralising antibodies (Daniels* et al.*, 1983; Koel* et al.*, 2013; Skehel & Wiley, 2000), these substitutions can affect both virus receptor-binding and antigenicity and studies suggest that receptor-binding avidity can be a driving force for antigenic drift (Daniels* et al.*, 1984; Hensley* et al.*, 2009). The HA of the H3N2 IAV has evolved extensively since its emergence in 1968. This evolution has resulted in current viruses having reduced avidity for sialic acid (SA) analogues of the human receptor (α2,6-terminally-linked SA-containing carbohydrates) and an even weaker ability to bind to avian SA receptors (α2,3-terminally-linked SA-containing carbohydrates) (Lin* et al.*, 2012; Medeiros* et al.*, 2001; Nobusawa* et al.*, 2000; Yang* et al.*, 2015). Associated with these changes is the observation that current H3N2 viruses are difficult to adapt to propagation in hens’ eggs.

Egg-adaptive AA substitutions can obfuscate the choice of vaccine viruses. In several 2012–2013 season H3N2 CVVs (IVR-165, NIB-79, and X-217) derived from A/Victoria/361/2011 (Vic361), egg-propagation resulted in AA substitutions at the RBS and known antigenic sites (Webster & Laver, 1980; Wiley* et al.*, 1981) (Table 1, Fig. S1, available in the online Supplementary Material). During the 2012–2013 season low vaccine effectiveness was reported in Canada (Skowronski* et al.*, 2013), USA (Flannery *et al.*, 2013), and Europe (Valenciano & Kissling, 2013), which was proposed to be due to AA substitutions in the H3N2 vaccine component (IVR-165), as opposed to antigenic drift in circulating viruses (Barr* et al.*, 2014; Skowronski* et al.*, 2014). As the substitutions were suspected to have altered the virus antigenicity, the H3N2 strain was changed from egg-propagated Vic361 (Vic361e) to egg-propagated A/Texas/50/2012 (Tex50e) for inclusion in the 2013–2014 vaccine following WHO recommendations (Barr* et al.*, 2014). Egg-propagation of the Tex50e CVV, X-223, resulted in AA substitution at position 226 in HA1, a residue frequently associated with changes in receptor-binding properties (Matrosovich* et al.*, 2000; Naeve* et al.*, 1984; Rogers* et al.*, 1983; Wan & Perez, 2007). Due to the emergence of viruses in new antigenically-distinct genetic clades, 3c.2a and 3c.3a, the H3N2 component for inclusion in the 2015 Southern Hemisphere vaccine was recommended to be changed to egg-propagated A/Switzerland/9715293/2013 (Switz13e) (WHO, 2014) and was also included in the Northern Hemisphere 2015–2016 season vaccine (WHO, 2015). Generation of Switz13e CVVs, X-247 and NIB-88, resulted in AA substitutions at position 219 (Table 1, Fig. S1).

Here we report on the effects of egg-adaptive substitutions in HA of recent H3N2 vaccine viruses on SA receptor-binding using a recently developed surface biolayer interferom etry (BLI) assay (Lin* et al.*, 2012), along with haemagglutination inhibition (HI) assays, in order to characterise the correlation between receptor-binding and the antigenic properties of these viruses. Using the vaccine virus Vic361e, recommended for use in vaccines in 2012 and 2013, as a prototype, a panel of viruses was generated by reverse genetics (RG) in order to relate the effect of individual AA substitutions on both receptor-binding and antigenicity. We further examined the receptor-binding and antigenic properties of vaccine viruses recommended for 2014 and 2015. Although a recent study has used BLI and glycan arrays to examine the receptor-binding properties of baculovirus-expressed H3N2 HA proteins from seasonal vaccine components up until 2013 (Yang* et al.*, 2015), no complementary antigenic analysis was done and the viruses have continued to change in the intervening period and are highly likely to do so in future seasons. To our knowledge this is the first time that quantitative receptor-binding assays and HI assays have been used together to investigate the correlative effects on receptor-binding and antigenicity of defined AA substitutions in H3N2 CVVs.

## Results and Discussion

### Receptor-binding of H3N2 candidate vaccine viruses and egg-propagated RG viruses

BLI receptor-binding assays were carried out to determine the binding avidity of WT, CVV, or RG H3N2 viruses, representative of 2012–2013, 2013–2014, or 2015–2016 seasons (Table 1), to avian (3-SLN) and human (6-SLN) receptor-analogues.

**Fig. 1.**
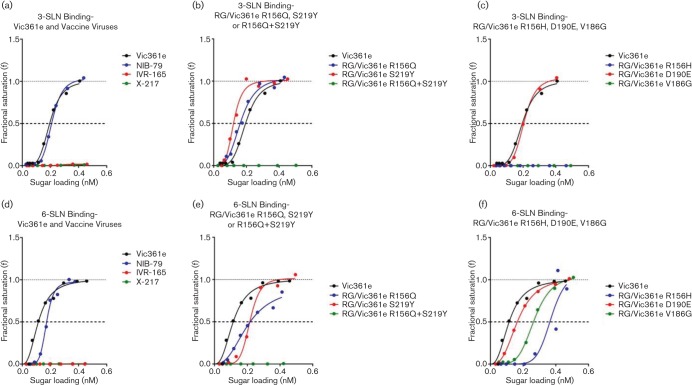
BLI receptor-binding assays of H3N2 viruses from 2012–2013 seasons to 3-SLN or 6-SLN receptoranalogues. Binding curves for egg-propagated Vic361e and CVV IVR-165, X-217, and NIB-79 to 3-SLN analogue (a) and 6-SLN analogue (d); binding curves for egg-propagated RG viruses bearing AA substitutions R156Q or S219Y or with the combined R156Q+S219Y substitutions compared to egg-propagated Vic361e for 3-SLN (b) and 6-SLN (e); binding curves for egg-propagated RG viruses bearing AA substitutions D190E or R156H or V186G compared to egg-propagated Vic361e for 3-SLN (c) and 6-SLN (f). Viruses were assayed at a concentration of 100 pM based on the nucleoprotein content of purified virus.

The recommended virus from which CVVs were produced for the Northern Hemisphere season 2012–2013 was A/Victoria/361/2011 (Vic361). Three CVVs were produced from V361e: IVR-165, X-217 and NIB-79 (Table 1). In the set of 2012–2013 season CVV and RG viruses, NIB-79 demonstrated a similar avidity for 3-SLN (Fig. 1a) but a decreased avidity for 6-SLN (Fig. 1d) compared with Vic361e. In contrast, no measurable binding of alternative CVVs IVR-165 or X-217 to either analogue was observed (Fig. 1a, d). Both IVR-165 and X-217 carry substitutions R156Q and S219Y, which are not seen in Vic361e or NIB-79.

RG viruses were produced with either one or both of the R156Q and S219Y AA substitutions to define which was responsible for the lack of measurable binding to the receptor-analogues. Introduction of substitutions R156Q and S219Y independently into Vic361e HA resulted in some increased binding to 3-SLN (Fig. 1b), but decreased binding to 6-SLN (Fig. 1e) when compared to Vic361e. Introducing the R156Q+S219Y combination into Vic361e resulted in no detectable binding of the virus to either 3-SLN (Fig. 1b) or 6-SLN (Fig. 1e). These results indicate that this AA combination at these positions significantly reduces the virus binding avidity to human and avian receptor-analogues, and they confirm the data for IVR-165 and X-217 (Fig. 1a, d). This double substitution may have caused a reduction in binding overall to receptor-analogues but could be the result of an adaptation to specific receptors present in the egg allantoic cavity, which may be responsible for the high growth in eggs of the double-substitution virus. It is notable that paired HA substitutions in H1 subtypes at positions 190 and 225 have previously been reported to alter receptor-binding specificity (Matrosovich* et al.*, 2000; Stevens* et al.*, 2006) highlighting the importance of examining single and combinations of AA substitutions arising during the complex process of egg-adaptation.

CVV NIB-79 encoded a D190E substitution in HA1. RG/Vic361e D190E, constructed to correspond to NIB-79, showed a similar binding profile to 3-SLN (Fig. 1c) with a slightly decreased binding avidity for 6-SLN (Fig. 1f) when compared with Vic361e, as was seen with NIB-79 (Fig. 1a, d). D190E is a common egg-adaptive substitution and the AA at this position has been shown to be involved in the receptor-binding specificity of both H3 and H1 subtypes (Glaser* et al.*, 2005; Matrosovich* et al.*, 2000; Nobusawa* et al.*, 2000).

**Table 1.** AA residues of interest present in and around the HA RBS of H3N2 egg- or cell culture-propagated prototype and eggpropagated CVV (V) influenza viruses

Virus*	Amino acid position (H3 numbering)						Virus binding to BLI receptor analogues†	
	156	186	190	219	225	226	3-SLN	6-SLN
A/Victoria/361/2011 cell-propagated WT (Vic361c)	H	G	D	S	N	I	–	–
A/Victoria/361/2011 egg-propagated (Vic361e)	R	V	D	S	N	I	++	++
IVR-165^V^	Q	V	D	Y	N	I	–	–
X-217^V^	Q	V	D	Y	N	I	–	–
NIB-79^V^	R	V	E	S	N	I	++	+
A/Texas/50/2012 egg-propagated (Tex50e)	H	V	D	F	N	I	–	–
X-223^V^	H	V	D	F	N	N	++	–
A/Switzerland/9715293/2013 cell-propagated WT (Switz13c)	H	G	D	S	D	I	–	+++
A/Switzerland/9715293/2013 egg-propagated (Switz13e)	H	V	D	S	D	I	+++	+
X-247^V^	H	V	D	F	D	I	–	+
NIB-88^V^	H	V	D	Y	D	I	–	+

*HA sequences of prototype viruses and CVVs were obtained from the Global Initiative for Sharing All Influenza Data (GISAID) EpiFluÉ platform (http://platform.gisaid.org/epi3/).

†Arbitrary BLI binding avidities of viruses to 3-SLN or 6-SLN analogues. +++, Very strong binding; ++, strong binding; +, detectable binding/weaker binding; detectable binding.

Vic361e differs from the cell culture-propagated virus (Vic361c) at two AAs in the vicinity of the RBS, 156 and 186. Two RG viruses RG/Vic361e R156H and RG/Vic361e V186G were constructed to represent viruses with single substitutions. Both RG viruses failed to bind 3-SLN (Fig. 1c) and had the lowest avidities for 6-SLN compared with Vic361e and other RG viruses assayed (Fig. 1f). Residue 186 of HA1 is recognised for its role in cell- or egg-adaptation (Gambaryan* et al.*, 1999; Lu* et al.*, 2005, 2006) and 186G makes the virus more ‘cell-like’. 156H also makes the virus more ‘cell-like’ and this property is probably responsible for the loss of 3-SLN binding. These observations also correlate with reports that recent human H3N2 viruses have lost the ability to bind to α2,3-linked SAs and propagate most efficiently in MDCK-SIAT1 cells, which over-express α2,6-linked SAs, compared with standard MDCK cells (Lin* et al.*, 2012; Oh* et al.*, 2008).

**Fig. 2.**
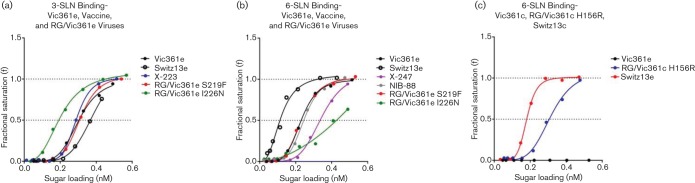
BLI receptor-binding assays of egg-propagated H3N2 viruses from 2013–2014 and 2015–2016 seasons and cell culture-propagated H3N2 viruses to 3-SLN and 6-SLN receptor-analogues. Binding curves for egg-propagated Switz13e, CVV X-223 and RG /Vic361e viruses with AA substitutions S219F or I226N to 3-SLN (2a) analogue compared with Vic361e; binding curves for Switz13e, its CVVs X-247 and NIB-88, and RG/Vic361e viruses with AA substitutions S219F or I226N to 6-SLN (2b) receptor-analogue compared with Vic361e; binding curves for exclusively cell culture680 propagated Vic361c and Switz13c, and RG/Vic361c virus bearing AA substitution H156R to 6-SLN receptor-analogue (2c). Viruses were assayed at a concentration of 100 pM based on the nucleoprotein content of purified virus.

In 2013, the H3N2 vaccine component recommendation changed from Vic361e to Tex50e. Tex50e showed no measurable binding to 3-SLN or 6-SLN (data not shown). CVV X-223, derived from Tex50e and carrying a substitution at residue 226 of HA1 (I226N), also failed to bind to 6-SLN (data not shown) but bound with high avidity to 3-SLN (Fig. 2a). The lack of binding to 6-SLN may be due to the maintained 156H in HA1, making the virus more ‘cell-like’ as described above for egg-propagated RG/Vic361e R156H (Fig. 1c). As demonstrated here, cell culture-propagated viruses encoding 156H also do not bind to 6-SLN (Fig. 2c). The AA substitution I226N carried by X-223 is probably responsible for the strong virus binding avidity for 3-SLN and AA substitution at position 226 of HA1 is a frequently documented host-receptor adaptation, with Q226L substitution causing altered binding preferences from avian to human receptors (Matrosovich* et al.*, 2000; Naeve* et al.*, 1984; Rogers* et al.*, 1983). In support of this, introduction of I226N into Vic361e HA to produce RG/Vic361e I226N resulted in increased binding avidity for 3-SLN (Fig. 2a) and decreased binding to 6-SLN compared with Vic361e virus (Fig. 2b). I226N substitution establishes a new potential glycosylation motif. Whether the motif is functional is not known, but it seems unlikely as the HA 220 loop is intimately involved in receptor-binding.

In September 2014, a change in the recommendation for the H3N2 component of the influenza vaccine was made due to the emergence of antigenically-distinct viruses in early 2014. A/Switzerland/9715293/2013 (Switz13e) was recommended as the prototype virus. In the set of 2015 and 2015–2016 CVVs derived from Switz13e, X-247 and NIB-88, both carried AA substitutions at residue 219, S219F and S219Y respectively, compared to the egg-propagated prototype Switz13e. Both bound to 6-SLN with low avidity compared to the Switz13e virus (Fig. 2b), and both failed to bind to 3-SLN (data not shown), unlike Switz13e which bound the avian receptor-analogue, albeit with low avidity (Fig. 2a). The observation that NIB-88 and X-247 failed to bind 3-SLN was surprising in light of the ability of both RG/Vic361e S219Y and RG/Vic361e S219F (Fig. 1b and Fig. 2a) to bind 3-SLN at high avidity. Tyrosine and phenylalanine contain an aromatic side chain, and are more hydrophobic than serine present in Switz13c and Switz13e. We speculate that this could affect the hydrophobicity of the HA molecule, and previous studies have indicated that differing hydrophobic states may affect antibody epitope binding and virus glycosylation profile (Long* et al.*, 2011; Pan* et al.*, 2011). Potentially, these substitutions at position 219 could also physically alter the RBS by affecting the 220-loop conformation due to the bulky, aromatic side-chain structures of tyrosine and phenylalanine. It is also possible that the phenotype of strong binding avidity to 3-SLN of viruses RG/Vic361e S219Y and RG/Vic361 S219F may be attributed to various potential combinations of AA residues at positions 219, 156 and/or 225, or additional substitutions located in regions other than those listed in Table 1 which may be compensating for the 219Y and 219F residue changes.

### Receptor-binding of H3N2 RG viruses propagated in cell culture

Both the exclusively cell culture-propagated cultivar of Switz13 (Switz13c) and the RG/Vic361c H156R virus bound with high avidity to 6-SLN (Fig. 2c) but no other cell culture-propagated viruses assayed (listed in Table 2) bound to 3- or 6-SLN even when 10 or 15 times more virus was used (data not shown). The introduction of H156R substitution into Vic361c made the RG virus more like the egg-propagated virus, which was found to bind with high avidity to 6-SLN (Fig. 1b, d, f). Results for cell culture-propagated cultivars of Tex50, RG/Vic361c D190E and RG/Vic361c I226N could not be generated as they propagated to very low yield, making subsequent analyses impossible.

**Table 2.** HA titres of egg- and cell culture–propagated prototype, CVV and RG viruses

Virus*	1 % Guinea pig RBC	0.5 % Turkey RBC	0.5 % Chicken RBC
A/Victoria/361/2011^E^	8192	16 384	2048
IVR-165^V^	524 288	131 072	131 072
X-217^V^	65 536	65 536	1024
NIB-79^V^	32 768	32 768	16 384
RG viruses derived from egg-propagated Vic361e			
RG/Vic361e R156Q^E^	131 072	524 288	131 072
RG/Vic361e R156H^E^	16 384	4	<
RG/Vic361e V186G^E^	1024	64	<
RG/Vic361e D190E^E^	8192	2048	4096
RG/Vic361e S219F^E^	131 072	16 384	4096
RG/Vic361e S219Y^E^	2048	1024	1024
RG/Vic361e I226N^E^	65 536	8192	32 768
RG/Vic361e R156Q+S219Y^E^	65 536	524 288	2048
RG/Vic361e R156H+S219Y^E^	65 536	8192	1024
A/Victoria/361/2011^C^	1024	<	<
RG viruses derived from cell-propagated Vic361c			
RG/Vic361c H156R^C^	256	32	<
RG/Vic361c H156Q^C^	1 048 576	16	<
RG/Vic361c G186V^C^	16 384	<	<
RG/Vic361c S219F^C^	32 768	<	<
RG/Vic361c S219Y^C^	16 384	<	<
RG/Vic361c H156Q+S219Y^C^	16 384	64	<
RG/Vic361c H156R+S219Y^C^	32	16	<
A/Texas/50/2012^E^	2048	512	128
X-223^V^	524 288	2 097 152	32 768
A/Switzerland/9715293/2013^E^	2048	1024	128
A/Switzerland/9715293/2013^C^	256	<	<
X-247^V^	32 768	16 384	2048
NIB-88^V^	32 768	16 384	4096

HA titres of purified virus initially diluted 50-fold in PBS, with 1 % guinea pig, 0.5 % turkey, or 0.5 % chicken RBC. <, No agglutination of RBC observed. *E, egg-propagated; C, cell culture-propagated; V, CVV. HA assays were repeated three times and the data presented are the modal values. Assays performed with RBC in PBS plus 20 nM oseltamivir carboxylate.

Failure of the majority of exclusively cell culture-propagated viruses to bind to receptor-analogues may be related to the AA at position 225. Previous BLI studies reported that representative H3N2 IAV collected between 2005 and 2010, propagated in cell culture, lost the ability to bind to 6-SLN by 2010. AA substitution D225N, occurring between 2004 and 2005, was demonstrated to be the major cause of a structural conformation change (Lin* et al.*, 2012). Vic361 and Tex50 retained 225N but the more recent Switz13 reverted to 225D and bound with high avidity to 6-SLN (Fig. 2c). However, introduction of substitution H156R into cell-propagated Vic361 (Vic361c) to produce RG/Vic361c H156R, also resulted in this virus binding with high avidity to 6-SLN (Fig. 2c) showing that substitution at position 225 is not the sole AA responsible for determining the binding profile. Along with residue 225, there were HA substitutions at several other positions in the more recently emerged 2014–2015 H3N2 viruses (WHO, 2015) which may contribute to the binding phenotype of Switz13 either singly or in combination. Residue 159 has been identified as being potentially responsible for the antigenic drift between 2013–2014 and 2014–2015 seasons (Chambers* et al.*, 2014), and due to its location on the HA1 molecule as a potential glycosylation site, it is also of interest in virus receptor-binding. Failure to bind 6-SLN might also relate to the physical composition of the receptor-analogue, either due to a low density or lack of presentation of modified types of α2,6-linked molecules. Binding studies using glycan microarrays recently demonstrated that binding of a baculovirus-expressed cell culture-propagated Vic361 HA protein was restricted to α2,6-linked receptor-analogues with increased glycan chain lengths (Yang* et al.*, 2015) which could make the receptor-analogue more accessible to the virus glycoprotein. This is supported by a recent review of glycan array screening data which suggests that since 1968 H3N2 IAV have generally lost the ability to bind to short branched glycans, and now bind preferentially to those with long linear backbone sequences (Air, 2014). Increased glycosylation of the HA1 globular head over the course of H3N2 circulation (Blackburne* et al.*, 2008; Cherry* et al.*, 2009; Kobayashi & Suzuki, 2012), presumably to escape neutralising antibodies (Skehel* et al.*, 1984), may also cause steric hindrance of binding to α2,6-linked analogues with short glycan chains.

### Agglutination of red blood cells (RBC) from different species by H3N2 viruses

RBC from different mammalian and avian species express cell-surface SAs of different linkages and modifications making them useful tools in indicating receptor-binding preferences of IAV (Ito* et al.*, 1997). Therefore, to complement the quantitative BLI measurements, HA assays were performed with guinea pig, chicken, turkey, and horse RBC. The results are shown in Table 2.

No cell culture-propagated viruses or the egg-propagated RG/Vic361e R156H were able to agglutinate chicken RBC, consistent with previous reports (Medeiros* et al.*, 2001; Nobusawa* et al.*, 2000). No viruses tested agglutinated horse RBC (data not shown). None of the cell culture-propagated viruses, with the exceptions of RG/Vic361c H156R and RG/Vic361c H156Q, which contain ‘egg-like’ residues at position 156, were able to agglutinate turkey RBC. These data correlate with the BLI binding data obtained for these viruses (Figs 1 and 2) and with previous reports of cell culture-propagated H3N2 IAV from 2005 onwards being unable to bind turkey RBC (Lin *et al*., 2012).

All viruses tested agglutinated guinea pig RBC which express more α2,6-linked than α2,3-linked SA molecules compared with chicken and turkey RBC (Lin* et al.*, 2012; Medeiros* et al.*, 2001; Thompson* et al.*, 2004). This included viruses which were unable to bind to 6-SLN in BLI assays, indicating that there may be certain types, densities, or structures of α2,6-linked molecules present on the RBC that are not fully represented by the SLN analogue. Similarly, CVVs IVR-165, X-217, NIB-88, and X-247 did not bind the 3-SLN analogue yet bound chicken and turkey RBC. Both BLI and RBC agglutination are good ways to show that changes in receptor-binding are occurring, however the BLI receptor-analogues are synthetic and the range of glycans on turkey and guinea pig RBC used in established influenza assays are not likely to be fully representative of the glycans found in the human host. This has already been shown for chicken RBC in studies using glycomics-based analyses which indicate that the N-glycan receptor profile on the surface of these RBC is not representative of the profile on the surfaces of human bronchial epithelial cells (Aich* et al.*, 2011), meaning that these glycans are unlikely to present the required virus-receptors for human IAV. The nature of sialylated glycan populations present on turkey and guinea pig RBC is currently unknown and further work to resolve this is warranted.

### Antigenic analyses of H3N2 RG viruses

To correlate the receptor-binding properties of the viruses analysed with their antigenic properties, HI assays were carried out on the panel of RG viruses, CVVs and cell culture-propagated and egg-propagated reference viruses (Table 3).

**Table 3.** HI titres of egg-propagated and cell-culture-propagated prototype, CVV and RG viruses and associated virus binding avidities to BLI receptor-analogues

	Haemagglutination inhibition titre^† ^ Post-infection ferret antisera					Virus binding to BLI receptor-analogues^‡^	
Test viruses*	Vic361e	Vic361c	NIB-79^V^	IVR-165^V^	X-217^V^	3-SLN	6-SLN
A/Victoria/361/2011^E^	**2560**	320	640	1280	1280	++	++
A/Victoria/361/2011^C^	640	**640**	160^§^	640	640	–	–
NIB-79^V^	1280	80^§^	**1280**	320	320	++	+
IVR-165^V^	2560	160	160^§^	**1280**	5120	–	–
X-217^V^	1280	160	320	2560	**1280**	–	–
RG viruses derived from egg-propagated Vic361e							
RG/Vic361e R156H^E^	1280	1280	640	2560	2560	– (↑)	+ (↓)
RG/Vic361e R156Q^E^	640	320	640	2560	2560	+++ (↑)	+ (↓)
RG/Vic361e V186G^E^	1280	1280	1280	1280	1280	– (↓)	+ (↓)
RG/Vic361e D190E^E^	1280	160	2560	320	320	++	+ (↓)
RG/Vic361e S219F^E^	1280	320	1280	640	640	++	++
RG/Vic361e S219Y^E^	2560	320	1280	1280	2560	+++ (↑)	+ (↓)
RG/Vic361e I226N^E^	1280	640	640	640	640	++	+ (↓)
RG/Vic361e R156Q+S219Y^E^	640	160	320	2560	2560	– (↓)	– (↓)
RG viruses derived from cell-propagated Vic361c							
RG/Vic361c H156R^C^	1280	320	640	640	640	–	++ (↑)
RG/Vic361c H156Q^C^	640	160	320	1280	1280	–	–
RG/Vic361c G186V^C^	2560	2560	1280	1280	1280	–	–
RG/Vic361c S219F^C^	640	640	320	320	320	–	–
RG/Vic361c S219Y^C^	640	320	320	640	1280	–	–
RG/Vic361c H156Q+S219Y^C^	640	160	320	1280	640	–	–
RG/Vic361c H156R+S219Y^C^	1280	160	640	1280	1280	–	–

*E, Egg-propagated; C, cell culture-propagated; V, CVV. †Homologous HI titres highlighted in bold text; HI titres decreased 4-fold compared to homologous titres highlighted in italic text, reductions of ≥8-fold marked with §. HI assays were repeated three times and the data presented are the modal values. Assays performed with guinea pig RBC in PBS plus 20 nM oseltamivir carboxylate. ‡Arbitrary BLI binding avidities of viruses to 3-SLN or 6-SLN analogues. +++, Very strong binding, (↑) increased binding compared with prototype; ++, strong binding; +, detectable binding; (↓), weaker binding compared with prototype; –,l no detectable binding.

Most viruses were recognised at titres within 2-fold of the homologous titre (2560) for antiserum raised against Vic361e, indicating antigenic similarity. However, 7 of the 19 test viruses were recognised at 4-fold reductions in titre (HI titres of 640) compared with the homologous titre of the antiserum, which is classed as being antigenically distinguishable from the homologous virus, but not enough to be defined as antigenically distinct. These viruses included Vic361c and RG viruses with AA substitutions at HA positions 156 and 219, RG/Vic361e R156Q, R156Q+S219Y, RG/Vic361c H156Q, S219Y, S219F, H156Q+S219Y. Thus reduced recognition by the antiserum raised against Vic361e can be associated withAA substitution at positions 156 and 219.

Seven out of 19 viruses tested with antiserum raised against cell culture-propagated Vic361c gave HI titres that were significantly reduced (160) compared to the homologous titre (640), and CVV NIB-79 was found to be poorly recognised by this antiserum at an 8-fold reduction in HI titre (80) indicating it was antigenically distinct from Vic361c. NIB-79 contains a D190E substitution, also shared with RG/Vic361e D190E, but not present in any of the other viruses tested. RG/Vic361e D190E showed reduced recognition by the antiserum (4-fold reduced over the homologous titre) indicating that it may be responsible for the change in antigenicity.

Antiserum raised against NIB-79 had a high homologous titre (1280) and CVV IVR-165 and cell culture-propagated Vic361c were poorly recognised (160). Moreover, egg-propagated RG virus RG/Vic361e R156Q+S219Y and cell culture-propagated RG viruses RG/Vic361c H156Q, S219F, S219Y, and H156Q+S219Y have significantly lower HI titres of 320 when tested against NIB-79 antiserum. Antisera raised against either IVR-165 or X-217 recognised NIB-79 and RG/Vic361e D190E at titres of 320, 4-fold reduced compared to the homologous titre.

RG/Vic361e I226N reacted well (HI titre 1280) with antiserum raised against Vic361e (homologous titre 2560), indicating that the CVV X-223 is antigenically similar to the Tex50 virus (Table S1) from which it was derived. Similarly, the I226N substitution has previously been reported to improve virus replication in eggs of a cold-adapted H3N2 IAV A/Brisbane/10/07 without affecting virus antigenicity (Chen* et al.*, 2010). It is worth noting that the effect of the I226N substitution has the potential to differ between RG/Vic361e HA and X-223 HA, as a single substitution in one virus background will not necessarily confer the same effect when introduced into another background, even if the differences between the two backgrounds are minimal.

The emergence of H3N2 antigenic variants necessitated a new vaccine component to replace Tex50e for the 2015–2016 season unlike the change in recommendation from the Vic361 CVV IVR-165, to Tex50e and the CVV X-223, which was due to egg-adaptations as a result of CVV propagation (Barr* et al.*, 2014).

It is noteworthy that our data (summarised in Tables 2 and 3, Figs 1 and 2) demonstrate that AA substitutions at position 156 create viruses that are less antigenically similar to the prototype with marked alterations in receptor-binding avidity and specificity. This includes the egg-propagated viruses RG/Vic361e R156Q, R156Q+S219Y, and cell-propagated viruses RG/Vic361c H156Q, and H156Q+S219Y which were all engineered to be partial analogues of CVVs IVR-165 and X-217. Reduced vaccine efficacy has previously been described for viruses with substitutions at HA1 position 156 (Kodihalli* et al.*, 1995). Although RG/Vic361e D190E and CVV NIB-79 reacted poorly in HI assays, there were no dramatic alterations in binding profile using BLI or RBC agglutination. From this we can deduce that the D190E substitution mainly affects antigenicity with only little effect on receptor-binding. In contrast, viruses carrying substitutions at HA1 position 186, notably RG/Vic361e V186G and RG/Vic361c G186V, showed significant alterations in BLI receptor-analogue and RBC binding with no major changes in antigenic properties. G186V is one of the most commonly identified egg-adaptations in H3N2 viruses and our data complement other studies that have also demonstrated that G186V substitution does not affect virus antigenicity. For example, this has been reported previously for H3N2 IAV circulating during the 2003–2004 season whereby introduction of HA1 substitution G186V improved virus replication in eggs but showed no altered antigenic properties (Lu* et al.*, 2005, 2006) and G186V in combination with N246K has been shown to significantly enhance virus propagation in eggs without changing virus antigenicity or immunogenicity of zoonotic H3N2 variant IAV CVV A/Indiana/08/2011 (Barman* et al.*, 2015). Furthermore, G186V in combination with L194P demonstrated increased immunogenicity in ferrets and higher homologous HI titres with H3N2 viruses from 2005 and 2007 (Chen* et al.*, 2010). The effects of substitutions at residue 219 are perplexing: cell-propagated viruses RG/Vic361c S219F and S219Y reacted less well in HI assays with 4-fold decreases in titre; however, egg-propagated viruses with the same substitutions, RG/Vic361e S219F and S219Y reacted well, and it is noteworthy that RG/Vic361e S219Y had an increased binding avidity for 3-SLN. It is possible that the AA substitutions at these two positions (186 and 219) act synergistically to alter virus antigenicity. The cell-propagated viruses, RG/Vic361c S219F and S219Y, reacted less well in HI assays and carried 186G while the egg-propagated-viruses, RG/Vic361e S219F and S219Y, reacted well in HI assays and carried 186V.

Due to the overlapping nature of the antibody and receptor-binding regions of the HA molecule, studies such as these are required to indicate which substitutions mainly affect antigenicity (D190E) or receptor-binding properties (V186G and G186V) or both, as seen in viruses with HA1 position 156 substitutions.

## Conclusion

The HA of H3N2 IAV has evolved continuously over the last 47 years resulting in changes in receptor-binding preference and avidity, and antigenic properties. Despite this, H3N2 viruses show no decrease in their ability to circulate within the human population (Appiah* et al.*, 2015; Broberg *et al.*, 2015; Gulati* et al.*, 2013).

SA receptor-binding properties of H3N2 IAV have been studied extensively using a variety of techniques which our data complement. However, BLI technology can quantify the effects of individual AAs on influenza receptor-binding. Here this quantitative assay has been used alongside HI assays to provide correlative data on receptor-binding and antigenicity of influenza CVVs recently recommended for inclusion in seasonal influenza vaccines.

Alterations in binding avidity affect the quantity of virus that gives a standardised number of HA units and therefore can influence the amount of antibody required for neutralisation in the HI assays. These data are used by the WHO to develop recommendations on the composition of influenza vaccines. Currently there is not enough clear information available as to how antigenic and non-antigenic factors (e.g. receptor-binding avidity) contribute to HI titres, as discussed in (Ndifon, 2011). As it is clear that one property can affect the other it is important to collect data about both antigenicity and receptor-binding of IAV during surveillance to better inform selection of variants for consideration as vaccine candidates.

The 2014–2015 influenza season was more severe than the previous season, with H3N2 viruses circulating as the dominant subtype (Appiah* et al.*, 2015; Broberg *et al.*, 2015). The UK Office of National Statistics has estimated that 43, 900 excess winter deaths occurred in England and Wales in the winter of 2014–15, the highest number since 1999-00, with respiratory diseases being the underlying cause of death in more than a third of all excess winter deaths (Office for National Statistics, 2015). Clearly, the importance of global influenza surveillance remains high. Information on the effect of individual and combinations of AAs present at defined positions in HA should be used discerningly when choosing future potential vaccine viruses, and is likely to be of great importance during selection of sequences for use in influenza vaccines that may be generated using new technologies such as gene synthesis (Dormitzer, 2015).

## Methods

### Cells. 

MDCK-SIAT1 cells engineered to over-express α2,6-sialyltransferase (Matrosovich* et al.*, 2003) were provided by M. Matrosovich (Marburg, Germany) and maintained in DMEM (Cat.No.D6429, Sigma) supplemented with 10 % heat-inactivated foetal calf serum, 100 µg mL^-1^ penicillin-streptomycin (Cat.No.P4333, Sigma) and 1  mg mL^−1^ G418 Sulphate (Cat.No.11811–031, Life Technologies) at 37 °C, 5 % CO_2_.

### Viruses.

Details of viruses used (A/Victoria/361/2011 (Vic361), A/Texas/50/2012 (Tex50), A/Switzerland/9715293/2013 (Switz13), and CVVs IVR-165, NIB-79, X-217, X-223, X-247, and NIB-88) are listed in Table S2.

### Reverse genetics viruses.

An eight-plasmid reverse genetics rescue system (Hoffmann* et al.*, 2000) was used to generate a panel of mutants carrying HA AA substitutions found in CVV, egg-, and cell culture-propagated H3N2 viruses (Table 4). HA and NA genes of cell and egg-propagated Vic361 were amplified using specific PCR primers (sequences available upon request) and cloned into vector pHW2000. Mutagenesis and transfection of 293T cells was performed as previously described (Lin* et al.*, 2012). MDCK-SIAT1 cells or the allantoic cavity of hens’ eggs were inoculated directly with supernatant from transfected 293T cells for RG virus recovery.

**Table 4.** Panel of cell culture-propagated or egg-propagated influenza A (H3N2) viruses created by reverse genetics All viruses contained six-segment background of A/Puerto Rico/8/1934 (PB1, PB2, PA, NP, M, NS1) plus mutagenized HA and prototype NA segments from Vic361. Egg-propagated Vic361e HA carries AA 156R and 186V, and cell culture-propagated Vic361c HA carries AA 156H and 186G.

Background virus details	Reverse genetics HA mutant
A/Victoria/361/2011(E) HA+NA	R156Q
	R156H
	V186G
	D190E
	S219Y
	S219F
	I226N
	R156Q + S219Y
A/Victoria/361/2011(C) HA+NA	H156R
	H156Q
	G186V
	S219Y
	S219F
	H156Q + S219Y
	H156R + S219Y

### Virus propagation and quantification.

Cell culture-propagated viruses were cultivated in MDCK-SIAT1 cells (Lin* et al.*, 2012) and egg-adapted viruses were propagated in the allantoic cavity of 10 to 11 day old embryonated hens’ eggs (Benton* et al.*, 2015) as previously described. Virus HA and NA sequences were confirmed by Sanger sequencing after each passage and before assays were carried out. Virus concentration was determined by solid-phase ELISA using a mouse monoclonal anti-nucleoprotein antibody (Lin* et al.*, 2012).

### Haemagglutination and haemagglutination inhibition assays.

Haemagglutination assays were performed with suspensions of 1 % (v/v) guinea pig (B&K Universal, Hull, UK), 1 % horse, 0.5 % chicken (both TCS Biosciences, Buckingham, UK), or 0.5 % turkey (Centre for Infections, Public Health England, London, UK) red blood cells (RBC). HI assays were performed using WHO recommended methods (WHO, 2011) in the presence of 20 nM oseltamivir carboxylate with 1 % (v/v) guinea pig RBC. CVV, prototype, and RG viruses were tested against a panel of post-infection ferret antisera obtained from stocks held by the Crick Worldwide Influenza Centre, The Francis Crick Institute, Mill Hill Laboratory. Assays were performed in the presence of 20 nM oseltamivir carboxylate (Roche, UK) to circumvent potential agglutination by the virus neuraminidase (Lin* et al.*, 2010).

### Receptor-binding assays using biolayer interferometry.

Virus binding to polyacrylamide-linked, 20 % (w/w) carbohydrate, polyvalent receptor-analogues, α2,3-sialyl lactosamine (3-SLN) or α2,6-sialyl lactosamine (6-SLN) (Lectinity Holdings, Russia), was measured using BLI assays on an Octet® RED platform (ForteBio, Pall Corp., a division of Pall Life Sciences, USA) as previously described (Lin* et al.*, 2012). Equilibrium measurements of virus binding were plotted as a function of amount of sugar immobilised on the biosensor calculated from the response amplitude during the sugar-loading step.

## Supplementary Data

268 Supplementary File 1
