# Supplementary material for: Effects of egg-adaptation on receptor-binding and antigenic properties of recent influenza A (H3N2) vaccine viruses
Source: J Gen Virol. 2016 Jun 15;97(6):1333–44. doi: 10.1099/jgv.0.000457 (PMC5394856; doi:10.1099/jgv.0.000457)
Supplement: Supplementary file 1 [file jgv-97-1333-s001.pdf]

Supplementary Figure 1. Representative structure of IAV (H3N2) receptor-binding site labelled with key amino acid residues in complex with human receptor-analogue (6-SLN)

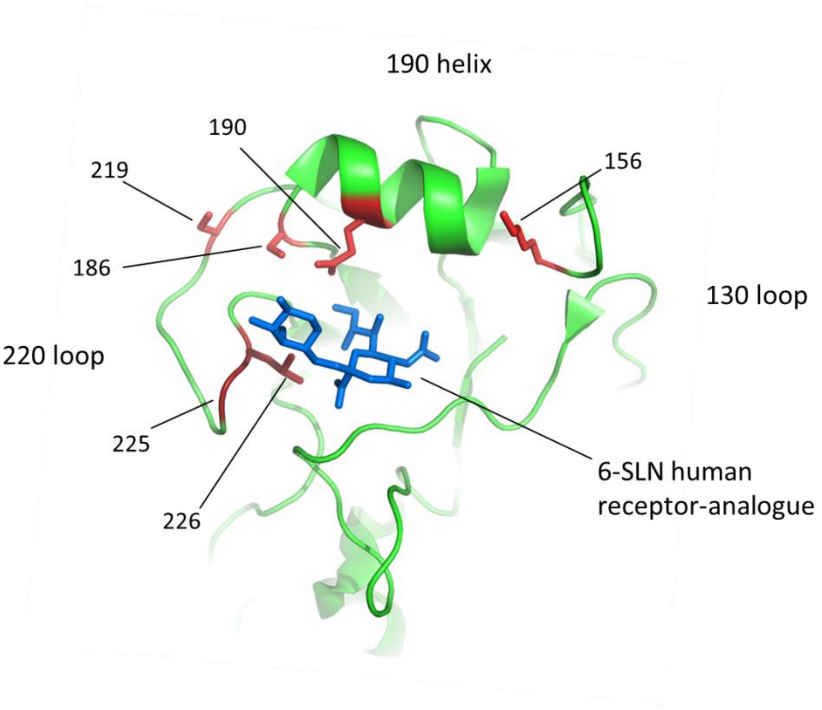

Monomeric structure of receptor binding site of representative influenza A (H3N2) virus HA in complex with a human receptor-analogue 6-SLN coloured blue (RCSB Protein Data Bank file reference 2YPG). Residues of interest in these studies; 156, 186, 190, 219, 225 and 226, are coloured red and labelled with amino acid residue number (H3 numbering). Diagram created using PyMol (Schrödinger).

Supplementary Table 1. HI Titres of egg-propagated Tex50e and CVV viruses

Haemagglutination Inhibition Titre† Post-infection Ferret Antiserum	
Test Viruses*	Tex50e
A/Texas/50/2012[E]	**5120**
X-223[V]	5120
X-223a[V]	2560

*E – egg-propagated and V – CVV. †Homologous HI titre highlighted in bold text.

Supplementary Table 2. Source of viruses and CVV used in these studies

Virus	Passage History	Originating Laboratory
A/Victoria/361/2011 egg-propagated	E3/E5	VIDRL, Pete Doherty Institute, Melbourne, Australia
A/Victoria/361/2011 cell-propagated	MDCK2/SIAT5	VIDRL, Pete Doherty Institute, Melbourne, Australia
IVR-165 CVV	Ex/E1	VIDRL, Pete Doherty Institute, Melbourne, Australia
X-217 CVV	Ex/E1	New York Medical College, USA
NIB-79 CVV	Ex/E1	NIBSC, UK
A/Texas/50/2012 egg-propagated	E5/E2	CDC, Atlanta, USA
X-223 CVV	Ex/E1	New York Medical College, USA
A/Switzerland/9715293/2013 egg-propagated	E4/E2	The Francis Crick Institute, Mill Hill Laboratory, London UK
A/Switzerland/9715293/2013 cell-propagated	SIAT1/SIAT5	The Francis Crick Institute, Mill Hill Laboratory, London UK
NIB-88 CVV	Ex/E1	NIBSC, UK
X-247 CVV	Ex/E1	New York Medical College, USA

All viruses were obtained from stocks held in the Crick Worldwide Influenza Centre, The Francis Crick Institute, Mill Hill Laboratory, UK. All CVVs are classical 6:2 reassortants containing HA and NA gene segments of the prototype virus with the remaining six genes from A/Puerto Rico/8/34, with the exception of X-247, which also contains the NS gene of prototype A/Switzerland/9715293/2013 making it a 5:3 reassortant. All CVVs were propagated in 10-11 day old embryonated hens' eggs. Other viruses were initially isolated from clinical isolates by National Influenza Centres before propagation in MDCK-SIAT1 cells or 10-11 day old embryonated hens' eggs by the originating laboratories. The HA and NA gene sequences of all viruses were confirmed by Sanger sequencing methods after propagation.
